# Intoxication after Extreme Oral Overdose of Quetiapine to Attempt Suicide: Pharmacological Concerns of Side Effects

**DOI:** 10.1155/2009/371698

**Published:** 2010-01-03

**Authors:** C. Müller, H. Reuter, C. Dohmen

**Affiliations:** ^1^Institute of Pharmacology, University of Cologne, Gleuelerstr. 24, 50931 Cologne, Germany; ^2^Department III of Internal Medicine, University of Cologne, Kerpenerstr. 62, 50937 Cologne, Germany; ^3^Max Planck Institute for Neurological Research, Gleuelerstr. 50, 50931 Cologne, Germany; ^4^Department of Neurology, University of Cologne, Gleuelerstr. 50, 50931 Cologne, Germany

## Abstract

Quetiapine is an atypical antipsychotic approved for the treatment of patients with psychotic disorders. Since approvement several case reports about intoxication with quetiapine were linked mainly with tachycardia, QT_c_-prolongation, somnolence, and hyperglycemia. Here, we present the first case report of an intoxication with an extreme overdose of quetiapine (36 g), ingested by a 32-year-old female (62 kg bodyweight) to attempt suicide. Symptoms associated with intoxication were coma without arterial hypotension, persistent tachycardia, hyperglycemia, and transient hypothyreoidism. QT_c_-interval was moderately extended. Management consisted of intubation for airway protection, gastric lavage, the use of activated charcoal, i.v. saline, and observation for 17 hours on an intensive care unit. Despite the extremely high dose of quetiapine, the patient recovered completely without residual symptoms.

## 1. Introduction

Quetiapine is indicated and approved for the treatment of psychotic disorders in adults by the U.S. Food and Drug Administration. The efficacy of quetiapine in the treatment of acute mania and depression associated with bipolar disorder and the therapy of schizophrenia with quetiapine has been proved in randomized controlled trials (for review, see [1]).

Pharmacokinetics and pharmacodynamics of quetiapine fumarate (Seroquel), a dibenzothiazepine antipsychotic agent bearing structural similarity to clozapine and olanzapine, have previously been described and reviewed [2–4]. After oral administration the drug is eliminated with a mean terminal half-life of approximately 7 hours [1]. The volume of distribution and total protein binding is 10 l/kg and 83%, respectively (Prod. Info Seroquel, 2004a). The drug is primarily metabolized by sulfoxidation and oxidation with the CYP3A4 isoenzyme and the 7-hydroxylated as well as the N-dealkylated metabolite are pharmacologically active [2].

Quetiapine shows affinity for multiple neurotransmitter receptors in in vitro binding studies. The drug exhibits high affinity for serotonergic 5-HT_2A_-receptors and moderate affinity for dopamine D_2_-receptors; whereas antagonism of D_1_- and 5-HT_1A_-receptors is relatively weak. The antipsychotic effect is explained by the antagonistic effect on D_2_-receptors and 5-HT_2A_-receptors [5].

Appreciable affinity for *α*_1_-adrenergic, *α*_2_-adrenergic, and histamine H_1_-receptors has also been observed [6].

Adverse effects even at the beginning of the therapy like sedation and somnolence are explained by antagonism of histamine H_1_-receptors. Orthostatic dysregulation, hypotension, and tachycardia are associated with an antagonistic effect on *α*_1_-adrenergic receptors. Quetiapine has also been reported to have an antagonistic effect on M_1_-muscarinic receptors resulting in anticholinergic mediated tachycardia [1, 7].

Quetiapine and other atypical antipsychotic drugs like clozapine or olanzapine provide antipsychotic efficacy with a lower risk of EPS compared to typical antipsychotics, but atypical antipsychotics are associated with a variety of metabolic and cardiovascular adverse effects particularly with regard to an overdose [8–15]. If treating patients with quetiapine one should be familiar with the clinical symptoms of quetiapine intoxication and its treatment. Here, we report on clinical effects of an extreme quetiapine overdose and how such an intoxication can be survived under appropriate treatment.

## 2. Case Report

We report on a 32-year-old female (62 kg bodyweight) with history of paranoid-hallucinatory schizophrenia and depression who was discovered by her companion in life 26 hours after ingestion of 36 g quetiapine (120 tablets of 100 mg and 80 tablets of 300 mg) and a suspected abuse of lorazepam. A previous suicide had been attempted 10 years before with haloperidol. Her past medical history was negative for cardiac dysrhythmia, hypertensive heart disease or a thyroid dysfunction. The emergency medical service found the patient comatose with response only to deep painful stimuli (Glasgow Coma Scale of 9), normotensive (127/83 mmHg), hyperglycemic capillary glucose level: 8.96 mmol/L = 160 mg/dl and tachycardiac with sinustachycardia of 140 bpm, capillary oxygen saturation was 98% and respiratory rate 12/min. There was no evidence of trauma or infection. During the transport to the hospital 40 mg of furosemide and 1000 mL saline were administered intravenously for detoxification and prophylaxis of hypotension. Vital signs were stable during transport.

On admission on the intensive care unit (ICU) the patient's condition worsened and she was intubated for airway protection. At this time venous blood gas on 30% FiO_2_ revealed pH of 7.38, pO_2_ of 45.3 mmHg, pCO_2_ 42.2 mmHg, HCO_3_^−^ 24.4 mmol/L, base excess −0.1 mmol/L, Hb 13.8 g/dl, K^+^ 3,7 mmol/L, Na^+^ 140 mmol/L.

To prevent her from further absorption of quetiapine a gastric lavage was performed (no pill fragments of ingested tablets were recovered) and the patient enterally received 25 g of activated charcoal with 5 g Glauber's salt (sodium sulfate) every 3 to 4 hours until dejection of black stool.

Laboratory data on admission including serum electrolytes, liver, and renal function parameters and blood count remained normal except glucose of 8.12 mmol/L (145 mg/dl), CK of 333 U/L (control value remained unchanged) with no evidence of myocardial insufficiency or infarction, CRP of 32 mg/L (control value 8 hours later increased to 76 mg/L), TSH 6.0 mU/L (control value 8 hours later increased to 8.56 mU/L), and leucocytosis with 12.06 (×10^9^/L).

The electrocardiogram at 36 hours postingestion revealed a moderately extended QT_c_ interval measuring 436 millisseconds (at the upper limit of normal) compared to QT_c_ interval of 388 ms after complete recovery. QT_c_-intervals were calculated using the Bazett formula [16].

A comprehensive drug screen via immunoassay was positive for tricyclic antidepressants and benzodiazepines in particular, although these results could not be confirmed by UV-HPLC-methods.

One day after admission cardiac and pulmonary status was stable (without signs of QT_c_-prolongation, arrhythmia, or tachycardia), state of consciousness improved, so that she could be extubated and was transferred to psychiatric crisis ward within 67 hours after ingestion of quetiapine. The increased levels for glucose and TSH lasted for 2.5 days, CK turned to normal after 6 days and CRP after roughly two weeks, respectively (see Table 1).

Serum-samples for pharmacokinetic analysis were collected directly on arrival in the emergency department, 5 hours after admission and the following two days (four samples in total). Quetiapine serum concentrations were determined by a validated UV-HPLC-method (linearity range 0.03–4.00 mg/L, lower limit of quantification (LLOQ) 0.03 mg/L, limit of detection (LOD) 0.01 mg/L). Drug concentrations and patient's data (gender, age, weight, height) as well as the ingested amount of quetiapine were processed by a commonly used pharmacokinetic program Mw/Pharm 3.50 [17]. Pharmacokinetic parameters were investigated based on an open two-compartment model and a Bayesian fitting procedure.

Elimination half-life was calculated as follows:

– *t*_1/2_ = ln 2/*λ*_z_ (elimination, *λ*_z_ is the rate constant corresponding to the terminal elimination).

**Figure 1:**
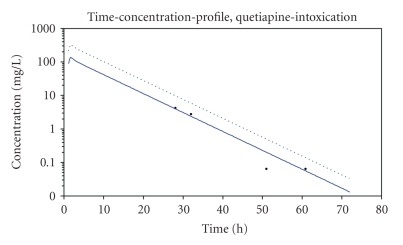
Serum time-concentration profile of quetiapine after ingestion of an assumed amount of 36 g Seroquel in a 32-year-old female patient. Solid-line: fitted line based on a two-compartment model assuming a reduced resorption. Dotted line: fitted line expected by population pharmacokinetic parameters and normal resorption.

Figure 1 exemplifies the obtained quetiapine concentration levels and the software generated time-concentration profile based on a two-compartment model (solid-line). In addition, the expected time-concentration profile based on population-pharmacokinetic data is shown (dotted line). Absorption was supposed to be reduced because of anticholinergic effects of quetiapine on intestinal tract.

The elimination half-life was *t*_1/2_ = 5.3 hours and was calculated based on the four obtained quetiapine concentration levels (c_1_ = 4.22 mg/L; c_2_ = 2.77 mg/L; c_3_ = 0.06 mg/L; c_4_ = 0.06 mg/L) with regard to patient's medical records and displayed a moderately increased elimination compared to population pharmacokinetic parameters. Additionally, the absorption rate constant k_a_ (time)^−1^ had to be adjusted and displayed a reduced absorption of quetiapine after ingestion. In this case the patient was supposed to have ingested 36 g quetiapine corresponding to a dosage of 580 mg/kg quetiapine. Volume of distribution was within the expected range (V_d_ = 9 L/kg based on fitted solid line, Figure 1).

Expected peak quetiapine concentrations for population-pharmacokinetic data were higher than the fitted time-concentration profile based on measured quetiapine concentrations and a two-compartment model.

## 3. Discussion

This is the first case report of an intoxication with such an extremely high amount of quetiapine (580 mg/kg). Remarkable side effects were tachycardia, somnolence, hyperglycemia, transient hypothyroidism, and a moderately prolonged QT_c_-interval, but the patient survived and fully recovered.

*Side effects: *tachycardia is most likely explained by an anticholinergic effect of quetiapine [7].

Symptoms of central nervous depression (somnolence, drowsiness) are primarly mediated through antagonistic effect of quetiapine on serotonergic 5-HT_2A_- and moderate antagonistic effect on dopamine type 2 D_2_-receptors. Prolonged tachycardia is usually explained by an antagonistic effect of quetiapine on *α*_1_-receptors [7]. Generally, the adverse effects like tachycardia, hyotension, and somnolence associated with quetiapine in particular after ingestion of an overdose can be explained by blockage of the alpha-adrenergic, muscarinic, and histamine receptors [1].

In recent publications surprisingly no statement about additional blood glucose laboratory data was provided, although several case reports after ingestion of high dosages of quetiapine were presented [7, 9].

The mechanism for hyperglycemia may be related to 5-HT_2_-receptor antagonism, which is associated not only with weight gain but also with hyperglycemia [18, 19]. 5-HT_2_-receptor antagonists can significantly decrease insulin sensitivity, an effect possibly mediated by the suppression of 5-HT_2A_-receptor-mediated glucose uptake in skletal muscle [8, 20]. For instance, 5-HT_2C_ knockout mice develop insulin resistance and impaired glucose tolerance and show severe weight gain [21]. This may be a likely mechanism for hyperglycemia in the therapy with atypical antipsychotics and in case of quetiapine overdose.

Moreover antipsychotic drug affinity and antagonistic effects on muscarinic receptors are significant predictors of the development of diabetes. Muscarinic M_3_-receptors are highly expressed by pancreatic *β*-cells and antagonism leads to dysregulation of glucose-dependent acetylcholin modulation of insulin secretion [22].

Suppression of compensatory insulin release may explain hyperglycemia in the present case report of quetiapine overdose.

Summarized, three receptor mediated mechanisms are commonly supposed for glucosedysregulation in particular after intoxication with an atypical antipsychotic.

First, 5-HT_2A_-receptor antagonism: suppression of receptor-mediated glucose uptake in skeletal muscle.

Second, 5-HT_2C_-antagonism is correlated with an increased risk of diabetes and weight gain most likely explained by 5-HT_2C_-receptor mediated insulin resistence and impaired glucose tolerance [21].

Third, M_3_-receptor-antagonism: dysregulation of glucose-dependent acetylcholin modulation of insulin secretion.

**Hypothyroidism**

Hypothyroidism associated with quetiapine treatment has been reported in previous studies under treatment with a normal daily dosage of quetiapine [23–26]. Although the mechanism is not known a competitive metabolism of thyroid hormones and quetiapine by UDP-glucoronyltransferase has been suggested as a plausible mechanism for a decrease of thyroid hormones during quetiapine treatment [25].

In case of a patient with compromised thyroid function receiving treatment with quetiapine may develop hypothyroidism and clinicians should consider routine monitoring of thyroid function [23].

*Observed Quetiapine Concentrations and Pharmacokinetics*. In our case report the measured serum concentrations of quetiapine were lower than the expected quetiapine concentrations due to the absorption rate constant, k_a_ (time)^−1^ and displayed a reduced absorption of quetiapine after ingestion (Figure 1). These findings are consistent with previously findings [11, 12]. Lower than expected peak concentrations may be explained by potential anticholinergic effects of quetiapine reducing absorption. With regard to a delayed absorption the expected time to maximum quetiapine concentration is supposed to be prolonged in these cases. In contrast to the findings of Hunfeld et al. we determined a serum time-concentration profile lower than the expected one. We assume that the impacts of side effects after ingestion of a very high amount of quetiapine may have contributed a decreased absorption with no influence on terminal elimination half-life.

With regards to the rescue case protocol the patient is supposed to have ingested 36 g quetiapine (rescue operation record: 120 tablets of 100 mg and 80 tablets of 300 mg). Although it cannot be ruled out that the ingested amount might have been less than 36 g, the time-concentration profile in this case is based on reduced absorption.

In contrast to these explanations the reason for considerably lower than expected quetiapine concentrations could obviously be an ingestion of a much lower amount of about 18 g quetiapine.

A comprehensive drug screen via immunoassay was positive for tricyclic antidepressants and benzodiazepines in particular.

Though an additional spectrophotometric (UV)-HPLC-method performed by the institute of forensic medicine did not confirm the concomitant intake of lorazepam.

Drug screening also was positive for tricyclic antidepressants, although the use of immunoassays is limited, because different drugs of the corresponding group may have quite different cross reactivity and/or pharmacological potency [27].

A comprehensive drug screening of tricyclic antidepressants could not be confirmed in additional analyses with UV-HPLC.

*Recommendations for Management of Therapy after Quetiapine Intoxication*. The treatment of quetiapine intoxications in case of an unknown ingested amount in particular consists in a supportive therapy and the patients should be monitored in an intensive care unit.

*Forced Diuresis with Furosemide and Saline*. Although 40 mg furosemide were administered during transport, the treatment with diuretic drugs for the purpose of enhanced elimination is no longer recommended [28]. Moreover the efficiency of this treatment is limited due to a relatively large volume of distribution of quetiapine (V_d_ = 10 l/kg) and a small renal elimination rate of quetiapine (less than 5%).

In case of a hypotensive patient the administration of diuretic drugs (e.g., furosemide) is not recommended, because of worsening the patients condition (further decrease of blood pressure).

*Gastric Lavage after 27 Hours*. To prevent further absorption of quetiapine a gastric lavage was performed and the patient was given activated charcoal with Glauber's salt 27 hours after quetiapine ingestion according to the recommendation of Burns et al.

At this time gastrointestinal passage of quetiapine is almost completed, although charcoal and glauber's salt (sodium sulfate) should be administered as soon as possible, if lag-time of drug ingestion is unknown. It should be noted that the risk of death following atypical antipsychotic overdose is very low [13] and gastric lavage is not routinely recommended after the 60 minutes time range [13, 29].

Monitoring of cardiac and respiratory function, intravenous access, and a 12-lead ECG is required [7, 13, 14].

Adminstration of intravenous magnesium sulfate (2 g bolus followed by an infusion of 2–4 mg/minute) is the intial therapy of choice regardless of serum magnesium level to the patient with QT_c_ prolongation. Serum potassium should be maintained in the high-normal range (4.5–5 mmol/L) and ingested drugs as well as interfering drugs with its metabolism have to be discontinued [30].

Monitoring of thyroid function in quetiapine treated patients with a history of or a vulnerability to thyroid disease is strongly recommended [31].

## 4. Summary

To our knowledge, the present case report is the first with an extremely high ingestion of quetiapine (36 g). Symptoms associated with intoxication were coma without arterial hypotension, persistent tachycardia, hyperglycemia, transient hypothyroidism, and a moderately extended QT_c_-interval. Management of overdose consisted in primarily supportive therapy on an intensive care unit.

In addition the present case may help us to understand the different side effects of an atypical antipsychotic drug with regard to receptor-pharmacological mechanisms.

Therapeutic drug monitoring and pharmacokinetic analysis of quetiapine after intoxication displayed a moderately increased elimination and a reduced absorption of quetiapine.

Despite the extreme overdose of quetiapine the patient exhibited a rapid clinical improvement and recovered without residual symptoms.

## Figures and Tables

**Table 1:** 

Parameter	Time after ingestion of quetiapine								
	26 h	28 h	29 h	32 h	37 h	51	62 h	140 h	331 h
Laboratory data:									
Quetiapine, *μ*g/L		4220		2770		64	64		
Na^+^, mmol/L			137		140		141	138	138
K^+^, mmol/L			3.6		4.6		4.0	3.6	4.3
Cl^−^, mmol/L					123		116	105	105
Glucose, mmol/L	8.96		8.12		5.94		5.04	5.66	5.49
CK, U/L			333		317		355	69	66
Leucocytes (×10^9^/L)			12.06		6.61		6.23	6.21	5.46
CRP, mg/L			32		76		96	11	<3
Free T3, ng/L			2.8		2.1		2.6		
Free T4, ng/L			18.6		14.1		12.0		
TSH, mU/L			6.04		8.56		3.35		

Vital parameters:									
BP syst., mmHg	127		110		120		130	130	
BP diast., mmHg	83		70		80		70	70	
HR, beats/min	140		120		90		85	85	
RR, resp./min	12		12		8		8		
QTc [ms]					436		404		388

CK: creatin kinase, CRP: c-reactive protein, T3: triiodothyronine, T4: tetraiodothyronine, TSH: thyroid-stimulating hormone, BP: blood pressure, HR: heart rate, RR: respiratory rate, QTc: cardiac QT interval (at a standardized heart rate of 60/minute).
